# Case report: a peculiar glomerulopathy in a patient suffering from nephrotic syndrome

**DOI:** 10.1186/s12882-019-1478-8

**Published:** 2019-08-22

**Authors:** Fabian Wöstmann, Roman-Ulrich Müller, Heike Göbel, Thomas Benzing, Jan U. Becker, Malte P. Bartram

**Affiliations:** 1 Department II of Internal Medicine and Center for Molecular Medicine Cologne, University of Cologne, Faculty of Medicine and University Hospital Cologne, Cologne, Germany; 2 CECAD, University of Cologne, Faculty of Medicine and University Hospital Cologne, Cologne, Germany; 3 Institute of Pathology, University Hospital of Cologne, Cologne, Germany

**Keywords:** Nephrotic syndrome, Membranous Glomerulopathy, Microspheres, Podocyte infolding, Renal biopsy

## Abstract

**Background:**

Podocyte infolding glomerulopathy (PIG) is a rare histopathologic finding with global infolding of the podocytes into the glomerular basement membrane (GBM), accompanied by microstructures underneath. Described in 2002 for the first time, PIG was proposed as a new pathological entity in 2008 based on the largest case series so far. Yet all of the described cases derive from Asian countries. We report a case from Germany fulfilling the diagnostic criteria of PIG. Considering the scarcity of data on this entity especially in Western countries, collecting cases like ours and multicentric meta-analyses will be crucial to obtain a better understanding of PIG, its causes, clinical course and potential treatment options.

**Case presentation:**

A 56-year-old Caucasian woman with a history of rheumatoid arthritis (RA), no other comorbidities and no known renal disease was admitted to the hospital with acute kidney injury (AKI) and nephrotic syndrome. Physical examination was unremarkable except for anasarca. Renal ultrasound revealed no abnormalities. Laboratory and urine analyses were consistent with the nephrotic syndrome and renal failure. Serological studies regarding ANA, ANCA, anti-PLA2R autoantibodies, complement, virus infections, immunofixation and quantitative light chain analysis were unremarkable. A renal biopsy was performed. Light microscopic examination showed flattened tubular epithelium consistent with acute tubular damage, no infiltrates and unremarkable glomeruli except diffuse and global holes in the GBM (Fig. 1a) and negative staining for immunoglobulin heavy-chains, light-chains and complement split products. Electron microscopy revealed a rare correlate for these holes: global peculiar infolding of podocyte cytoplasm into the GBM. Most of these infoldings were accompanied by condensation of the GBM underneath. No such condensation or electron dense deposits were found without these infoldings or outside the GBM.

**Conclusion:**

Here we report the first case of PIG outside of Asia. Since there are only few reports about this specific finding, we feel there is a need to share information in an attempt to accumulate knowledge about this possible new entity and potential treatment options.

## Background

PIG is a rare and peculiar finding in kidney biopsies. The largest study up to date, conducted by Joh et al. in 2008 comprised 25 Patients, all from Japan [1]. Outside of Japan only two cases have been published by now, one in South Korea [2] and another from India [3].

Accompanying clinical findings and the age of onset show a wide variation. Most patients so far have been female and there was a noticeable association with autoimmune disorders, especially systemic lupus erythematosus (SLE) and other collagen diseases [1]. Also one case associated with multiple myeloma in a Japanese patient was described [4] The Japanese consensus recommends a clinical sub-classification depending on whether PIG is associated with collagen disease or not [1]. Accordingly, our Patient would be classified as PIG type A, collagen disease-associated.

Altogether, PIG appears to be a histopathological pattern common to a number of different causes. Up to now, there is discussion how this very rare nephropathological entity translates into a clinical diagnosis and what is the preferred treatment. As to the clinical course and light microscopic findings, PIG may be difficult to distinguish from certain forms of membranous nephropathy (MN) [5]. In our case both would be well in line with an atypical form of MN. However, neither immunohistochemistry nor EM revealed the typical immune complexes of MN.

We hope this case description, the first from a Western country, contributes to the understanding of this rare disease.

## Case presentation

A 56 year old obese Caucasian woman with a 12 year history of rheumatoid arthritis (RA), no other comorbidities and no known renal disease was transferred to our hospital with acute kidney injury and nephrotic syndrome.

The patient had been well until 11 days prior to admission when she experienced joint pain. She continued her regular RA medication with certolizumab and NSAID. Within the following days the patient noted oliguria and swelling of the face and legs. On admission, the patient presented well-oriented, with normal vital parameters (blood pressure 130/75 mmHg, heart rate 84/min) and a body weight of 87.9 kg (regular weight 82 kg). Physical examination was inconspicuous except for anasarka and an erythematous rash with papules in the epigastric region, appearing shortly after the self-administered dose of certolizumab.

Renal ultrasound revealed no abnormalities, there were no signs of renal vein thrombosis.

Laboratory results were consistent with the clinical picture of the nephrotic syndrome and showed an increased creatinine of 4.37 mg/dl, hypalbuminemia of 18 g/l and hypercholesterinemia (LDL 301 mg/dl). Urinary diagnostics showed glomerular proteinuria in the nephrotic range (albuminuria 6.2 g/g creatinine, erythrocytes 35 U/l). Serological studies regarding ANA, ANCA, anti-PLA2R autoantibodies, complement, viral testing (HBV, HCV, HIV, Hantavirus), serum/urine immunofixation and quantitative light chain analysis returned negative.

**Fig. 1:**
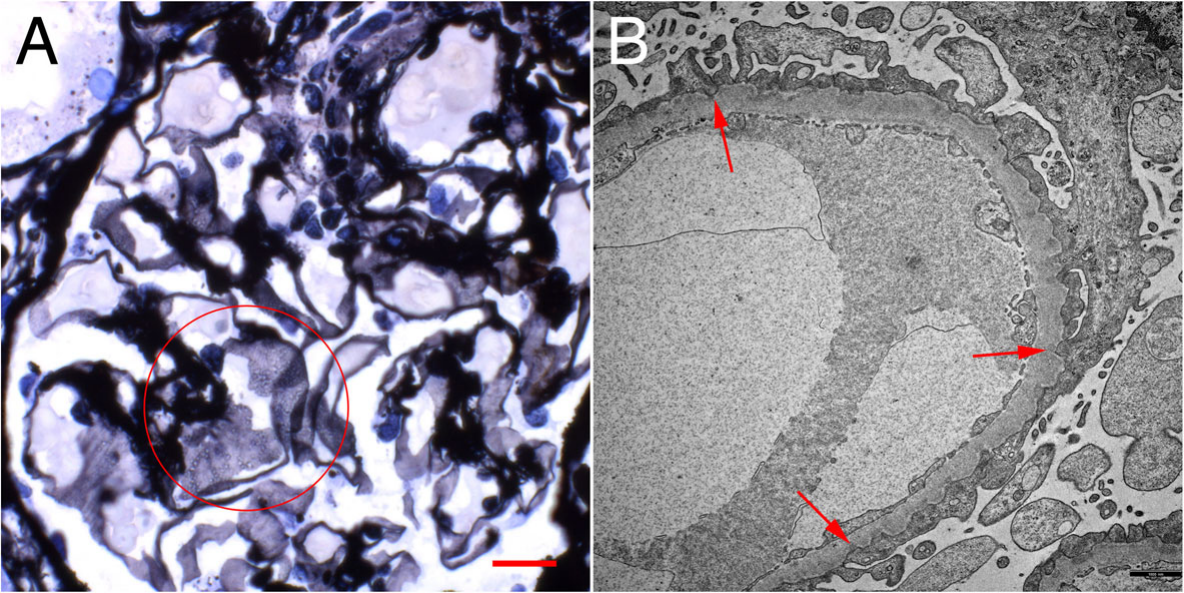
**a** Jones silver staining shows holes in the glomerular basement membrane (circle). Original magnification × 630, scale bar represents 50 μm. **b** Electron microscopy shows the ultrastructural correlate to these holes: infolding of the podocyte cytoplasm into the glomerular basement membrane, most of them with underlying densities (red arrows). This finding is characteristic for the podocyte infolding glomerulopathy (PIG). Transmission electron microscopy, original magnification × 10000, scale bar represents 1000 nm)

A renal biopsy was performed. Light microscopic examination showed flattened tubular epithelium consistent with acute tubular damage, no infiltrates and unremarkable glomeruli except diffuse and global holes in the glomerular basement membrane (GBM) on Jones’ silver stains (Fig. 1a) and negative staining for immunoglobulin heavy-chains, light-chains and complement split products. Electron microscopy (EM) showed the peculiar findings in Fig. 1b.

In most cases, holes in the GBM are indicative of membranous nephropathy (MN). However, MN was ruled out by negative immunohistochemistry. Instead, EM did reveal a different, exceedingly rare correlate for these holes: global peculiar infolding of the silver negative podocyte cytoplasm into the silver positive GBM [1]. Such infolding is not found in minimal change glomerulopathy. Most of these infoldings were accompanied by condensation of the GBM underneath. No such condensation or electron dense deposits were found without these infoldings or outside the GBM ruling out abortive forms of MN which can show only scant immunoglobulin and complement deposits. Moreover, the typical thinning, thickening and basket-weaving of Alport’s syndrome, an important differential diagnosis, were absent.

According to the Japanese consensus definition, these findings are diagnostic for PIG. The largest study up to date comprised 25 Patients from Japan [1]. Outside of Japan only two cases have been published [2, 3]. We could not find spheroid or tubular inclusions in the GBM which accompany the infoldings in PIG type B and exist without these infoldings in PIG type C. Thus our case would be classified as PIG type A.

Clinical findings and the age of onset show a wide variation. Most patients were female and there was an association with autoimmune disorders, especially systemic lupus erythematosus (SLE) and other collagen diseases [1].

Up to now, there is discussion how this very rare nephropathological entity translates into a clinical diagnosis and about the preferred treatment. As illustrated in our case, the clinical course and light microscopic findings of PIG may be difficult to distinguish from certain forms of MN [5]. However, neither immunohistochemistry nor EM revealed the typical immune complexes of MN. Based on the biopsy findings and clinical pattern we considered this case as a RA-associated nephropathy with PIG. Taking into account the good response in a similar case and the rheumatic disease [3], we discontinued certolizumab and started treatment with high dose prednisone and rituximab (2 × 1 g rituximab given 2 weeks apart), afterwards the steroids were rapidly tapered.

**Fig. 2:**
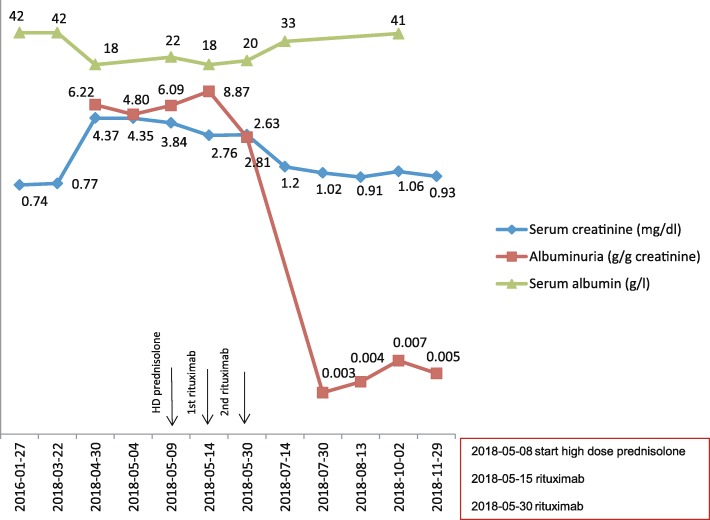
Graph showing the course of serum creatinine, albuminuria and serum albumin after onset of the disease and after initiation of the very effective treatment with prednisolone and rituximab. Note the logarithmic scale of the y-axis

After initiating this therapy, albuminuria (from 6.22 g/g to 2.63 g/g creatinine) as well as serum creatinine levels (from 4.37 mg/dl to 2.81 mg/dl) declined within 4 weeks. Two months later, there was no relevant albuminuria detectable and the serume creatinine levels remained stable around 0.9–1.0 mg/dl (Fig. 2). In addition, the patient had absolutely no complaints regarding her RA.

## Discussion and conclusions

So far most reports about PIG have speculated on the pathogenesis and etiology of the infolding and underlying microstructures accompanying the infoldings in PIG type B and C. Still there is no consensus about the origin of the infoldings or the underlying microstructures, animal models of PIG are still missing. Hepatitis B virus [6], complement activation [5], villous cytoplasmic protrusions of endothelial cells [1], or a reaction to podocyte injury secondary to a kidney injury [7] have been suggested as the cause of the microstructure formation in the GBM. Nevertheless, so far none of the above mentioned was proven to be a common feature of all the known cases of podocyte infolding.

Podocytic infolding per se can have several causes as shown by Masuda et al. in a series of 126 biopsies with membranous nephropathy [6]. 77.8% of the cases showed occasional podocytic infolding. However global and diffuse podocytic infolding as observed in our cases was very rare and only found in one of the 126 cases [6]. Certainly Alport’s syndrome is in the list of differential diagnoses. The clinical course in our patient and the lack of more typical findings argue against this differential diagnosis; in case of doubt genetic testing should be considered.

The patient described in this report presented with acute kidney injury. From the cases published so far, only around 25% had increased serum creatinine levels [1–4, 8]. In our case the intake of NSAIDs may have contributed to the development of AKI.

With the etiology and contibuting factors still unclear and a varied clinical presentation PIG should be considered a pattern of injury rather than an etiologically defined diagnostic entity, much like podocyte foot process effacement or focal and segmental glomerulosclerosis (FSGS). In line with the published literature we believe that the RA might be responsible – through an unknown mechanism – for the renal disease of our patient. Reviewing the published cases, none has been associated with a TNF-alpha inhibitor. Still – considering the scarcity of data – we cannot exclude a causal link. We hope that our case report will alert nehrologists and nephropathologists to this rare diagnostic entity and will stimulate larger case collections required to better define the etiology, diagnostic criteria, clinical associations, prognosis and treatment options for PIG.

Since many cases are connected to collagen and other rheumatic diseases we would like to emphasise the importance of a renal biopsy for these patients.

Regarding the literature most patients with the nephropathological pattern of a podocyte infolding glomerulopathy (PIG) in the kidney biopsy have been treated with steroids [1, 4, 7, 8], sometimes additional immunosuppressants like mycophenolate mofetil (MMF) or rituximab were utilised. To avoid long term high dose steroid treatments and considering the frequent coexistence of autoimmune diseases, it might be helpful to initiate a steroid sparing treatment early on. In our case the treatment with rituximab was very effective in reducing the proteinuria and improving the renal function as well as improving the rheumatoid arthritis.

## Data Availability

All the data relevant to this report are included in the manuscript.

## Abbreviations

AKI: Acute kidney injury
ANA: Antinuclear antibodies
ANCA: Anti-neutrophil cytoplasmatic antibodies
Anti-PLA2R: Anti-Phospholipase A2 receptor
FSGS: Focal and segmental glomerulosclerosis
GBM: Glomerular basement membrane
MMF: Mycophenolate mofetil
MN: Membranous nephropathy
NSAID: Nonsteroidal anti-inflammatory drugs
PIG: Podocyte infolding glomerulopathy
RA: Rheumatoid arthritis
SLE: Systemic lupus erythematosus
